# Supplementary material for: Machine learning partners in criminal networks
Source: Sci Rep. 2022 Sep 21;12:15746. doi: 10.1038/s41598-022-20025-w (PMC9492767; doi:10.1038/s41598-022-20025-w)
Supplement: Supplementary file 1 — Supplementary Information. [file 41598_2022_20025_MOESM1_ESM.pdf]

Machine Learning Partners in Criminal Networks

Diego D. Lopes[1], Bruno R. da Cunha[2,3], Alvaro F. Martins[1], Sebastián Gonçalves[4], Ervin K. Lenzi[5], Quentin S. Hanley[6], Matjaž Perc[7,8,9,10,†], and Haroldo V. Ribeiro[1,*]

[1]Departamento de Física, Universidade Estadual de Maringá – Maringá, PR 87020-900, Brazil
[2]Rio Grande do Sul Superintendency, Brazilian Federal Police – Porto Alegre, RS 90160-093, Brazil
[3]National Police Academy, Brazilian Federal Police – Brasília, DF 71559-900, Brazil
[4]Instituto de Física, Universidade Federal do Rio Grande do Sul – Porto Alegre, RS 91501-970, Brazil
[5]Departamento de Física, Universidade Estadual de Ponta Grossa – Ponta Grossa, PR 84030-900, Brazil
[6]School of Science and Technology, Nottingham Trent University, Clifton Lane, Nottingham NG11 8NS, United Kingdom
[7]Faculty of Natural Sciences and Mathematics, University of Maribor, Koroška cesta 160, 2000 Maribor, Slovenia
[8]Department of Medical Research, China Medical University Hospital, China Medical University, Taichung, Taiwan
[9]Alma Mater Europaea, Slovenska ulica 17, 2000 Maribor, Slovenia
[10]Complexity Science Hub Vienna, Josefstädterstraße 39, 1080 Vienna, Austria
[†]email: matjaz.perc@gmail.com
[*]email: hvr@dfi.uem.br

Supplemental Materials

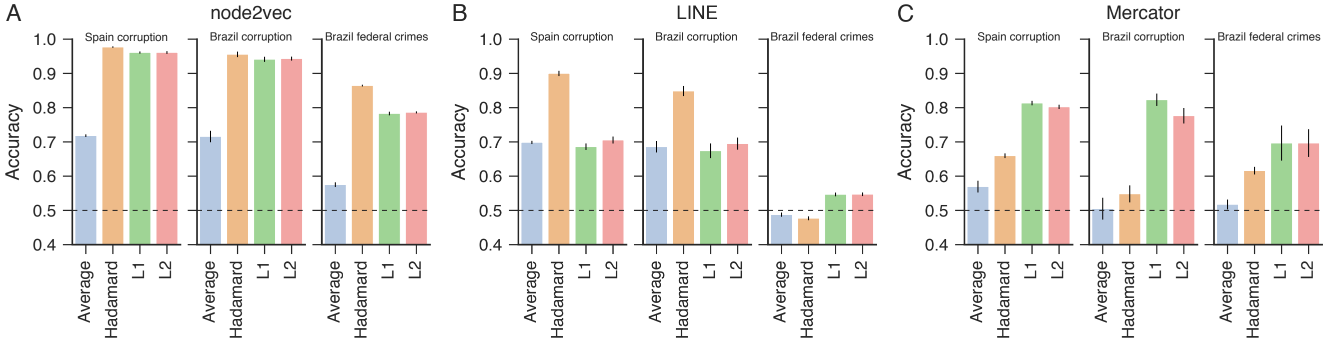

Figure S1. Average accuracy of logistic classifiers trained for predicting missing links in the Spanish corruption network, Brazilian corruption network, and Brazilian criminal intelligence network with (A) *node2vec*, (B) LINE, and (C) Mercator representations of nodes and different binary operators. The bars stand for the average accuracy in the test sets over ten replicas of the embedding and training processes (error bars represent one standard deviation). The test sets are generated by randomly removing 10% of network edges and sampling the same number of false connections. The horizontal dashed lines indicate the baseline accuracy.

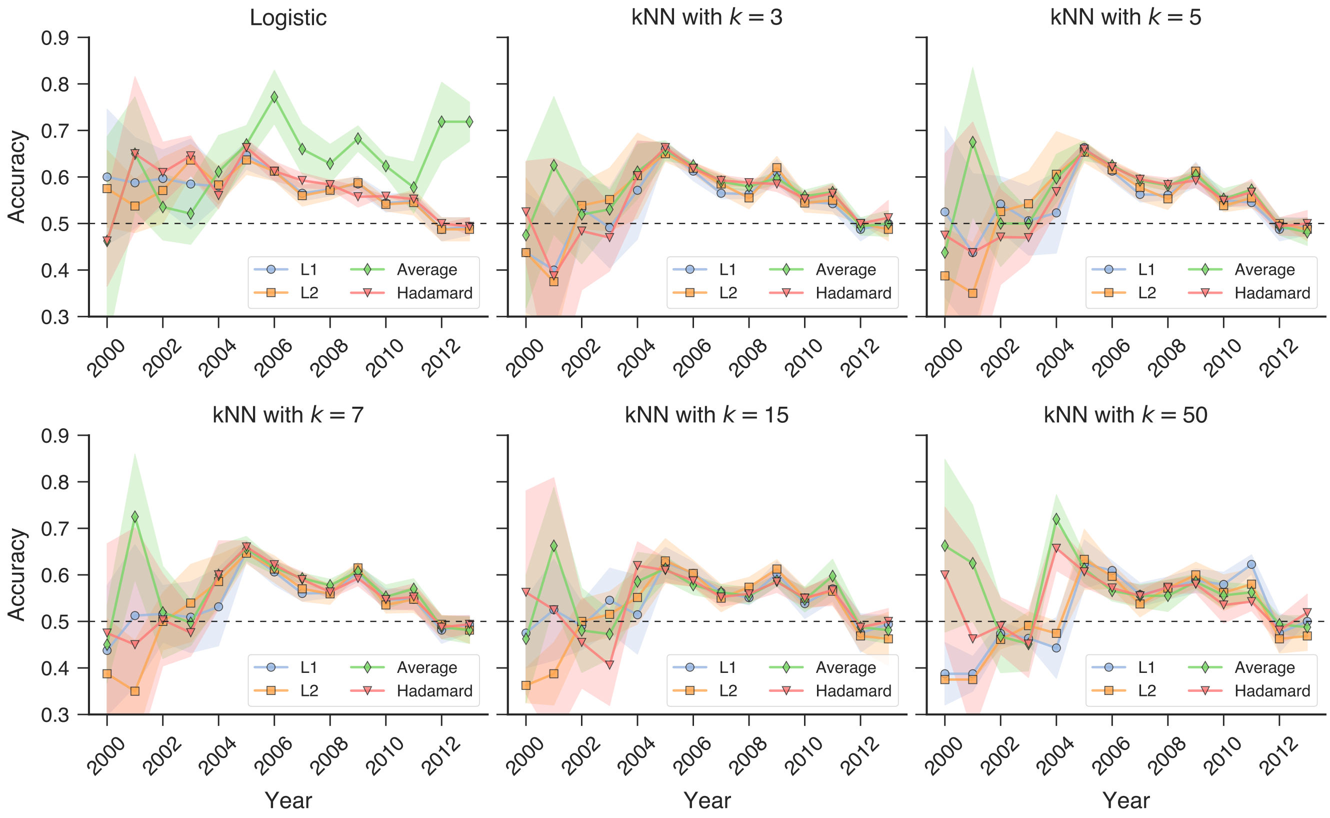

Figure S2. Average accuracy in tasks of predicting future partnerships evaluated in test sets of the Brazilian corruption networks as a function of the threshold year for different binary operators. The upper left panel shows the results obtained using logistic classifiers and all other panels depict the accuracy for kNN classifiers with different number of neighbors (k, shown above the panels). The markers represent the average accuracy on the test sets estimated from ten realizations of the embedding and training processes (shaded regions stand for one standard deviation band). The horizontal dashed line indicates the baseline accuracy.

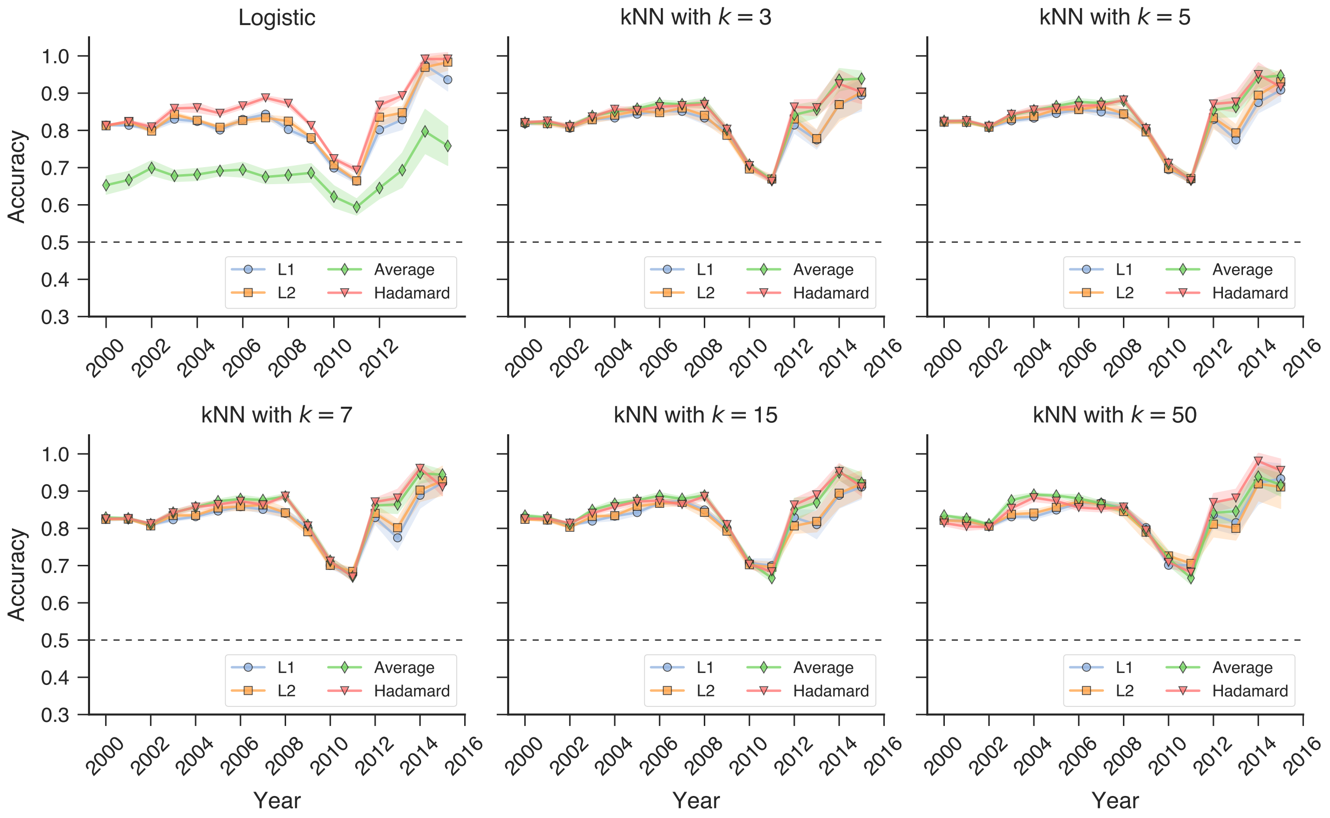

Figure S3. Average accuracy in tasks of predicting future partnerships evaluated in test sets of the Spanish corruption networks as a function of the threshold year for different binary operators. The upper left panel shows the results obtained using logistic classifiers and all other panels depict the accuracy for kNN classifiers with different number of neighbors (k, shown above the panels). The markers represent the average accuracy on the test sets estimated from ten realizations of the embedding and training processes (shaded regions stand for one standard deviation band). The horizontal dashed line indicates the baseline accuracy.
